# Printed Potentiometric Ammonium Sensors for Agriculture Applications

**DOI:** 10.1021/acsomega.4c05746

**Published:** 2024-11-20

**Authors:** Anju Toor, Payton Goodrich, Tyler L. Anthony, Claire Beckstoffer, Haeshini Jegan, Whendee L. Silver, Ana Claudia Arias

**Affiliations:** † School of Materials Science and Engineering, Georgia Institute of Technology, Atlanta, Georgia 30332, United States; ‡ Department of Electrical Engineering and Computer Science, University of California Berkeley, Berkeley, California 94720-1770, United States; § Department of Environmental Science Policy and Management, University of California Berkeley, Berkeley, California 94720-1770, United States

## Abstract

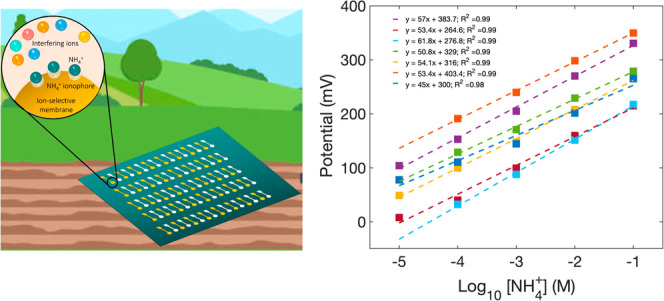

Ammonium (NH_4_^+^) concentration is critical to both nutrient availability and nitrogen (N) loss in soil ecosystems but can be highly variable across spatial and temporal scales. For this reason, effectively informing agricultural practices such as fertilizer management and understanding of mechanisms of soil N loss require sensor technologies to monitor ammonium concentrations in real time. Our work investigates the performance of fully printed ammonium ion-selective sensors used in diverse soil environments. Ammonium sensors consisting of a printed ammonium ion-selective electrode and a printed Ag/AgCl reference were fabricated and characterized in aqueous solutions and three different soil types (sand, peat, and clay) under the range of ion concentrations likely to be present in soil (0.01–100 mM). The response of ammonium sensors was further evaluated under variable gravimetric moisture content in the soil to reflect their reliability under field conditions. Ammonium sensors demonstrated a sensitivity of 53.6 ± 5.1 mV/decade when tested in aqueous solution, and a sensitivity of 55.7 ± 11 mV/dec, 57.5 ± 4.1 mV/dec, and 43.7 ± 4 mV/dec was measured in sand, clay, and peat soils, respectively.

## Introduction

Soil nitrogen (N) is a primary soil macronutrient and an important limiting factor for plant growth.^1^ Nitrogen can be found in many forms in soils, including nitrate (NO_3_^–^), ammonium (NH_4_^+^), nitrite (NO_2_^–^), organic N, and gaseous forms that include dinitrogen gas (N_2_), ammonia (NH_3_), nitric oxide (NO), and nitrous oxide (N_2_O).^2^ Nitrate and ammonium are the dominant forms of soil N biologically available for uptake by plants and thus are commonly used in agricultural fertilizers. Monitoring soil nitrate and ammonium ion concentrations is critical to managing agricultural systems, as it can improve fertilization efficiency and reduce environmental pollution from nitrogen saturation. In particular, elevated soil nitrogen levels can cause nitrate leaching, leading to the eutrophication of watersheds and groundwater contamination.^3,4^ In addition, saturation of bioavailable nitrogen can lead to an increase in soil emissions of particulate matter, which causes harmful human respiratory effects, and emissions of nitrous oxide, which is a potent greenhouse gas that contributes to climate change.^5,6^

Soil ammonium and nitrate concentrations are important and interacting drivers of nitrification and denitrification, both of which occur in agricultural soils. Nitrification rates are primarily controlled by the availability of ammonium and oxygen in soils, while denitrification rates are primarily controlled by the presence of denitrifying bacteria, reductants, nitrate ion concentration, and the absence of oxygen.^7^ Increased ammonium and nitrate ion concentrations through fertilizer addition have both been shown to lead to spikes in nitrous oxide production, increasing nitrous oxide emissions by an average of 216% across ecosystems.^8^ While the main drivers of nitrification and denitrification are well-known, continuous, in situ monitoring of soil ammonium and nitrate is required to better understand how spatial and temporal variation in these drivers will impact nitrous oxide emissions, particularly with the control and distributions of nitrous oxide “hotspots” and “hot moments,” large emission events that occur infrequently across time and space but contribute a significant proportion of the total soil nitrous oxide emissions.^9,10^

Specifically, ammonium monitoring has received increased attention as compared to nitrate due to reasons such as efficiency in plant uptake, better retention in soil, significant environmental impact, fertilizer efficiency, etc. To provide in situ, continuous, real-time reporting on soil ion concentrations across an agricultural field, deploying a network of miniaturized, low-cost, durable sensors that are sensitive and selective to respective soil ions will be necessary. Electrochemical sensors specifically potentiometric ion-selective sensors have demonstrated potential for miniaturization and accurate detection of nitrate in soil samples.^1,11,12^

A potentiometric ion-selective sensor measures the potential difference between an ion-selective electrode (ISE) and reference electrode (RE) under no current flow conditions. The potential of the ISE undergoes a significant change as the concentration of the target analyte changes. The RE is designed to maintain a constant potential in varying ionic environments. The ISE is based on an ion-sensitive polymeric membrane containing an ionophore, a reagent that selectively binds with the ion of interest.^13−19^ Polyvinyl chloride (PVC) is the most commonly used membrane matrix, and silver/silver chloride (Ag/AgCl) is used as a RE.^20^ The potential between the ISE and RE, *E*, varies with the activity, *a*_ion_ (or concentration *C*_ion_, for a dilute solution) of the ion of interest, according to the Nernst equation
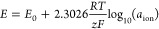
1where *E*_0_ is the standard potential, *R* is the ideal gas constant, *T* is the temperature, *z* is the ion charge, and *F* is the Faraday constant. The sensitivity (V/dec) is evaluated by
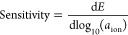
2The Nernstian response occurs when the response of an ISE is determined over a given range of concentrations of the target ion. According to the Nernst equation, at ambient temperature, the electrode will undergo a 59.1 mV step change in potential per factor of 10 change in the concentration of a monovalent ion (*z* = 1). Ammonium is a monovalent cation; hence, the anticipated sensitivity of the sensors is 59.1 mV/decade. In practice, every factor of 10 increase in ammonium concentration will result in a potential increase of 59.1 mV.

In the development of ammonium sensors for agricultural soil monitoring, rigorous testing for interfering ions stands as a critical step to ensure accuracy and reliability in measurements. The complexity of soil chemistry, characterized by a plethora of ions, such as potassium, sodium, and calcium, poses a significant challenge for the specificity of ammonium sensors. These ions, often present in concentrations comparable to or even higher than ammonium, can lead to erroneous readings if not adequately accounted for.^21,22^ Thus, the efficacy of an ammonium sensor in agricultural applications hinges not only on its sensitivity to ammonium but also on its ability to resist interference from these ubiquitous soil constituents.

**Figure 1:**
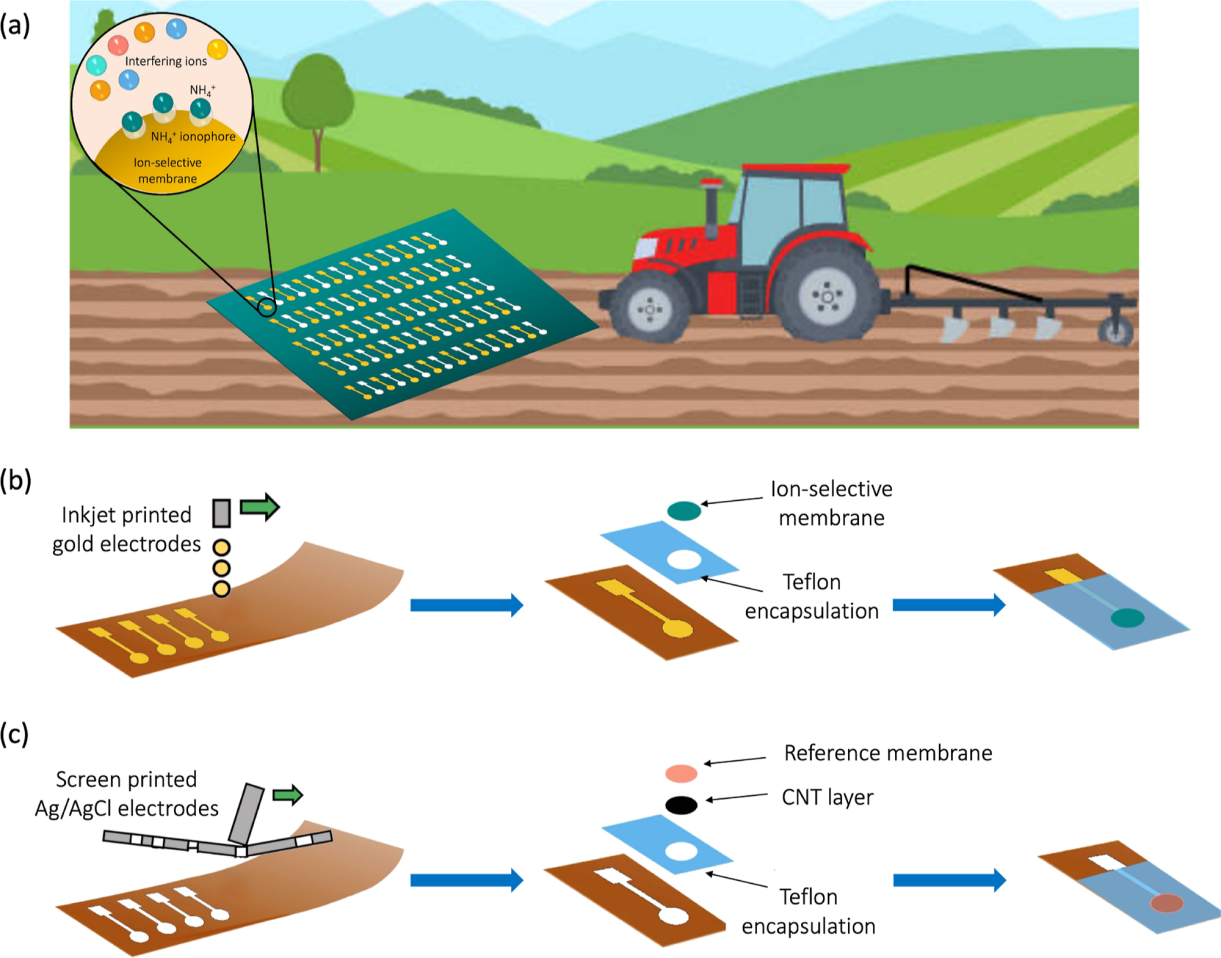
(a) Printed ammonium sensors could be deployed to map ammonium in an agriculture field. (b) Ammonium ISEs are made by inkjet-printing gold onto a substrate, followed by the lamination of a Teflon tape. The membrane solution is drop-cast on the exposed area of the electrode. (c) REs are made by screen-printing Ag/AgCl ink onto the substrate and then encapsulating it with Teflon. A carbon nanotube transducer layer is drop-cast, followed by the PVB/salt membrane.

To realize the complete benefits of printing, such as high-throughput manufacturing, low cost, easily customizable form factors, etc., fully printed ammonium sensors are reported. We envision that arrays of printed ammonium sensors would be deployed in the field at a high spatial density as shown in Figure 1a. Here, we report on the characterization of the selectivity and sensitivity of printed ammonium sensors in solution and soil. Printed Ag/AgCl REs and ammonium ISEs were fabricated and tested against a commercial RE followed by pairing of a printed Ag/AgCl RE with an ammonium ISE to obtain an ammonium sensor. Ammonium sensors have demonstrated high sensitivity and selectivity in solution; here, their performance in direct soil types is reported.

## Materials and Methods

### ISE Fabrication

Gold electrodes were printed on a 25 μm-thick polyethylene naphthalate substrate using a Harima Nanopaste (Au) NPG-J gold ink in a Dimatix inkjet printer at ambient conditions according to the process illustrated in Figure 1b. The desired pattern (3.5 mm diameter circle connected to a 1 mm wide trace) was generated in CAD software and converted to a format compatible with Dimatix software for waveform application. Printed gold electrodes were sintered at 250 °C for 50 min and then encapsulated with 75 μm-thick laser-cut Teflon tape with circular windows of 5 mm diameter for the active area. The window in the tape is larger than the electrode to allow space for the ion-selective membrane (ISM) to seal to the substrate, preventing bubbles or delamination of the membrane. ISM solutions were fabricated by mixing 0.2 wt % ammonium ionophore I, 69 wt % 2-nitrophenyl octyl ether, and 30.8 wt % PVC. A total of 0.5 g of this mixture was dissolved in 5 mL of tetrahydrofuran (THF). 6 μL of the membrane solution was drop-cast on the printed gold electrode surface. The resulting ISE was dried in a fume hood overnight, at room temperature.

### RE Fabrication

Ag/AgCl REs were screen-printed on 100 μm-thick poly(ethylene terephthalate) by using Engineered Materials Systems, Inc. CI-4001 ink as illustrated in Figure 1c. During screen printing, a silkscreen with a predetermined pattern is flooded with ink. The ink is then pressed through the screen onto the substrate below. The same pattern as the gold electrodes was used for printing Ag/AgCl REs. The printed Ag/AgCl electrodes were then cured at 120 °C in a vacuum oven for 2 h. Before testing, each RE was encapsulated with laser-cut Teflon tape 75 μm-thick.

A CNT transducer layer is deposited on top of the Ag/AgCl electrode.^23−25^ This transducer layer was composed of 0.01 g of CNT (iP-Single-Walled Carbon Nanotubes from Carbon Solutions, Inc.) and 0.05 g of F127 (poly(ethylene glycol)-*block*-poly(propylene glycol)-*block*-poly(ethylene glycol) diacrylate) dissolved in 10 mL of THF, which were sonified for 1 h in an ice bath using a Branson Digital Sonifier probe. The resulting mixture was deposited on the printed RE surface as 4 μL. The RE fabrication is finalized with the deposition of a reference membrane consisting of Butvar B-98 poly(vinyl butyral) (PVB) and sodium chloride (NaCl). The reference membrane was prepared by dissolving 1.58 g of PVB and 1.00 g of NaCl in 20 mL of methanol. This mixture was sonified for 30 min in an ice bath. The resulting mixture was drop-casted on the CNT transducer as 6 μL.

Further details of the fabrication process and the need for a CNT transducer and PVB + NaCl reference layer are discussed in our previous work.^26^ Briefly, introducing a CNT layer between the PVB + NaCl membrane and the Ag/AgCl layer resulted in an RE with minimal sensitivity to variations in the concentration of the NaCl solution.

For soil measurements, ammonium sensors, each consisting of a printed ISE and a printed RE, were mounted to an acrylic block for mechanical stability. 8331D silver conductive epoxy (MG Chemicals) was used to connect wires, and the joint was encapsulated by Gorilla two-part epoxy.

### Measurements in Solution

#### Sensitivity

Ammonium chloride (NH_4_Cl) was dissolved in deionized water and diluted to 0.01, 0.1, 1, 10, and 100 mM concentrations to perform sensitivity measurements. Open-circuit potentiometric measurements were performed using Ivium-*n*-Stat from Ivium Technologies B.V. Prior to measurement, electrodes were conditioned for at least 2 h in 100 mM NH_4_Cl. Commercial Ag/AgCl electrodes with the liquid filling solution were obtained from Millipore Sigma (Z113107).

#### Interference

Selectivity coefficients of the ammonium ISEs were evaluated using the separate solution method (SSM) according to the IUPAC recommendations.^27^ The ISEs were submerged in 0.1–100 mM solutions of CaCl_2_, KCl, MgCl_2_, Na_2_SO_4_, NaH_2_PO_4_, NaNO_3_, and NH_4_Cl, all obtained from Millipore Sigma. To better understand interference with nonchemical signals, the sensor response was also evaluated under varying light, temperature, and strain conditions, with the results shown in Figure S1 of the Supporting Information.

#### Stability

Current-reversal chronopotentiometry was performed to investigate ISE potential stability and determine the ISE’s bulk resistance and capacitance.^28^ A printed ISE was arranged in a three-electrode setup as the working electrode, a commercial Ag/AgCl electrode as the RE, and a platinum wire electrode as the counter electrode. After conditioning in a 100 mM NH_4_Cl solution, the printed ISE was polarized with a current of ±10 nA for 60 s each (120 s total), and the potential was recorded with the electrode immersed in 100 mM NH_4_Cl solution. The capacitance was extracted from the rate of potential change and the current input, while the bulk resistance of the electrode was calculated from the ohmic drop when the current was reversed.

To further evaluate the stability of the printed ISEs, open-circuit potentiometry was employed over a duration of 10 days. The printed ISE and a commercially available RE were immersed in 500 mL of 100 mM NH_4_Cl solution, and the potential difference between the ISE and the commercial RE was systematically recorded every 10 s. The average drift was determined by fitting a linear regression to the plot.

### Soil Measurements

Ammonium sensors were tested in three different soil types, namely, sand, clay, and peat. Sensor measurements and soil KCl extractions were conducted by using the same method across all soil types. Approximately 40 g of soil (sand density = 1.63 g/cm^3^, peat density = 0.60 g/cm^3^, and clay density = 1.07 g/cm^3^) was added to corresponding 200 mL jars per soil treatment. Six treatments were prepared by performing serial dilutions to create 1000, 100, 10, 1, 0.1, and 0.01 mM ammonium solutions. Approximately 40 mL of the corresponding solution was added to each jar (*n* = 6 replicates per soil type per solution) to create a soil environment at approximately 50% gravimetric moisture content.

Individual ammonium sensors were connected to a Campbell CR1000 (Campbell Scientific, Logan, Utah) datalogger. The soil was added to each beaker, followed by ISE and RE and the corresponding ammonium chloride solution. Sensors were inserted into each soil treatment, and readings were taken while the output voltage stabilized for ≥3 min per concentration. After measurement in one container, the sensor was removed, rinsed with deionized water, and inserted into the next container. After completion of the measurement, KCl extraction was conducted on each soil sample to determine the total extractable ammonium concentration in each soil treatment. To generate a 1:5 soil-to-KCl solution mass ratio, 15 g of each soil sample was added to approximately 75 mL of a 2 M KCl solution. These samples were then shaken for 1 h at 180 rpm. After shaking, the samples were filtered through prewashed Whatman 1 filter paper (Cytiva, Marlborough, MA). The extracts were frozen until colorimetric ammonium analysis using a Seal AQ300 Analyzer (Seal Analytical, Mequon, Wisconsin) could be done.

## Results and Discussion

**Figure 2:**
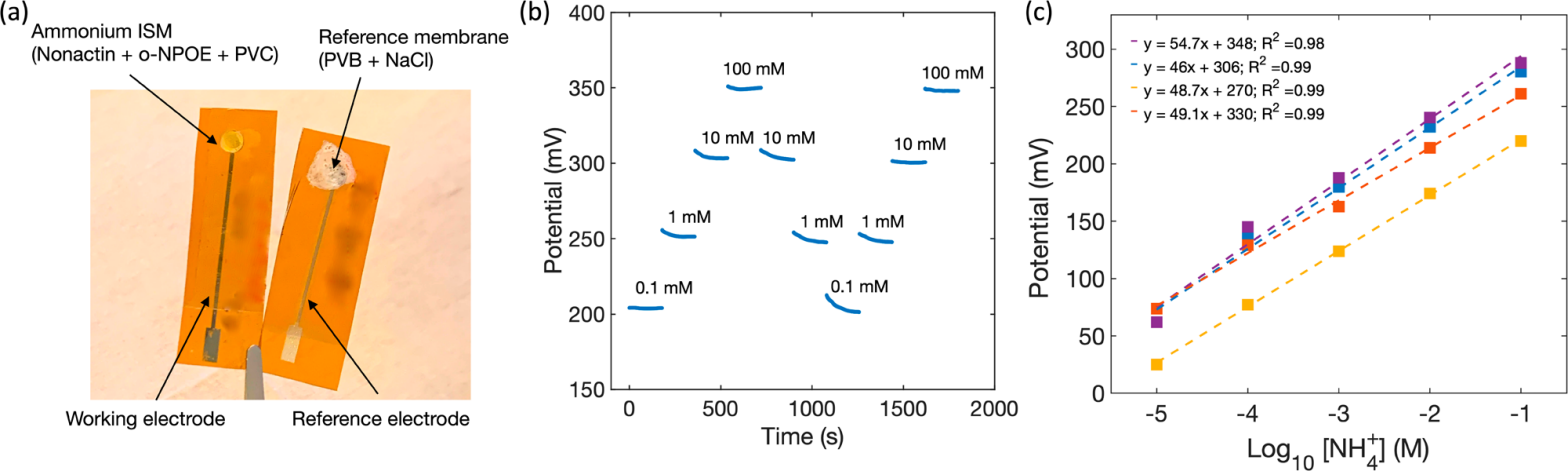
(a) Printed ammonium ISE and a printed Ag/AgCl RE. (b) Potential over time for an ammonium ISE measured against a commercial glass RE in NH_4_Cl solutions of varying concentrations. (c) Sensitivity plots for ammonium ISEs fabricated in multiple batches, showing near-Nernstian response of an average 49.6 ± 3.6 mV/decade.

The printed ISEs and REs were fabricated on separate substrates so that they could be characterized independently before pairing the electrodes to form a fully printed sensor. Figure 2a shows an ammonium sensor comprising a screen-printed Ag/AgCl chloride RE and an ISE comprising an inkjet printed gold electrode with an ammonium ISM. Before assembling the printed ammonium ISE and printed RE together to form a fully printed ammonium sensor, the performance of the ammonium ISE and printed RE was characterized separately against a commercial glass Ag/AgCl electrode. For these measurements, an ISE or RE was paired against the commercial RE, and the output potential was measured in ammonium chloride (NH_4_Cl) solutions at varying concentrations. First, we will discuss the performance of printed ammonium ISEs, followed by the characterization of printed REs, and fully printed ammonium sensors.

### Ammonium ISE

#### Sensitivity Measurement

The sensitivity of the printed ammonium ISEs is characterized by measuring the potential between a printed ISE and a commercial RE over time in 0.1–100 mM NH_4_Cl solutions. Figure 2b shows the potential over time response of a printed ammonium ISE measured in changing ammonium concentrations. Each flat step in Figure 2b represents an ammonium ISE being tested in a solution of a given ammonium concentration. A step change to a different potential value is observed as the sensor is transferred and tested in a beaker containing ammonium solution of a different concentration. The sensor output, potential, is plotted as a function of ammonium concentration in the log scale in Figure 2c. The sensitivity data for different sensors are offset from one from another. The variation in *E*_0_ observed in Figure 2c is common in ISEs and means that each sensor must be individually calibrated before use. The variation in *E*_0_ is attributed to the differences in phase boundary potentials at interfaces between the ISM/electron conductor and CNT transducer layer/reference membrane.^29^ Each dashed line is a linear fit to the potential recorded in the 0.01–100 mM range for each sensor. This measurement was repeated for four sensors fabricated in multiple batches, as indicated by the multiple linear fit lines shown in Figure 2c. The average sensitivity was calculated by taking the average of the slopes of the measured potential vs logarithm of the ion concentration (i.e., sensitivity) plot obtained for each sensor. The upper and lower limits correspond to the standard deviation from the average sensitivity. The average sensitivity for printed ammonium ISEs was 49.6 ± 3.6 mV/decade.

#### Interference Characterization

The interference of the ammonium ISEs was analyzed using the SSM for the most prevalent ions in soil.^27,30^ In the SSM, the potential of the ISE is measured in two different concentration solutions separately, where one contains the ion A with activity *a*_A_ (no B) and the other contains the ion B with the same activity *a*_B_ (no A) to attain the same measured potential. The selectivity coefficients were obtained by solving
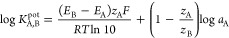
3where A denotes the primary cation (NH_4_^+^) and B denotes an interfering ion. The potentials *E*_A_ and *E*_B_ were calculated by performing a linear regression of each curve and solving for the potential at each *a*_A_ and then taking the average of the *K*_A,B_ calculated at each decade of concentration change. Ideally, the selectivity coefficient should be less than 1, and as it approaches zero, this indicates that the ISE is not sensitive to the interfering species under consideration. The selectivity coefficients were calculated from eq 3 and are reported in Table 1 as log *K*_A,B_^pot^. The most interfering ion observed was potassium (K^+^), which was expected due to the similarity of size and charge for ammonium and potassium ions. Calcium (Ca^2+^) and sodium (Na^+^) ions also demonstrated measurable interference, though at nearly 1.5 orders of magnitude less than potassium. Still, this indicates that in soils with high concentrations of K^+^, Ca^2+^, or Na^+^, the sensor response might be unreliable and may require site-specific calibration. These findings are in agreement with the literature.^22^

**Table 1:** Selectivity Coefficients for Selected Ions Obtained for Ammonium-Selective Electrodes

ammonium-selective electrode		
ion	slope ± SD.	log *K*_A,B_^pot^ ± SD.
Ca^2+^	36.033 ± 0.056	–2.364 ± 0.288
K^+^	45.995 ± 0.300	–0.820 ± 0.005
Na^+^	38.682 ± 1.495	–2.312 ± 0.007
NH_4_^+^	53.593 ± 0.324	N/A

#### Stability Characterization

One of the widely known challenges for printed ISEs is the susceptibility of their potential to drift over time or potential instability. Various mechanisms influence potential instability in printed ISEs, including electrode polarization, membrane fouling, water layer formation, and ionophore leaching, to name a few. For a printed ISE to be stable and reliable, it is ideal to have interfaces that are nonpolarizable, exhibit high exchange current densities, and have a large capacitance.^29^

Current-reversal chronopotentiometry was employed to investigate the susceptibility of the printed ISE to drift and calculate the capacitance and bulk resistance of the electrode. A current of ±10 nA was applied to the printed ISE to polarize the surface of the electrode, as shown in Figure S2a in the Supporting Information. The electrode resistance was measured to be 456.25 kΩ, and the low-frequency capacitance was measured to be 23.38 μF. These values are comparable to similar electrodes in the literature.^28,31,32^

The potential of the printed ISE vs a commercial Ag/AgCl RE in 100 mM NH_4_Cl was recorded over a 10 day period to further investigate potential stability, as shown in Figure S2b in the Supporting Information. The average drift was found to be −0.394 mV/h over the 10 day duration. For agricultural sensing application where crop cycles last months, a drift rate on the order of 1 μV/h or less is required to maintain a reasonable error.^33^ Thus, further improvements to reduce the steady-state drift are necessary for long-term use in agricultural soil.

### RE Characterization

**Figure 3:**
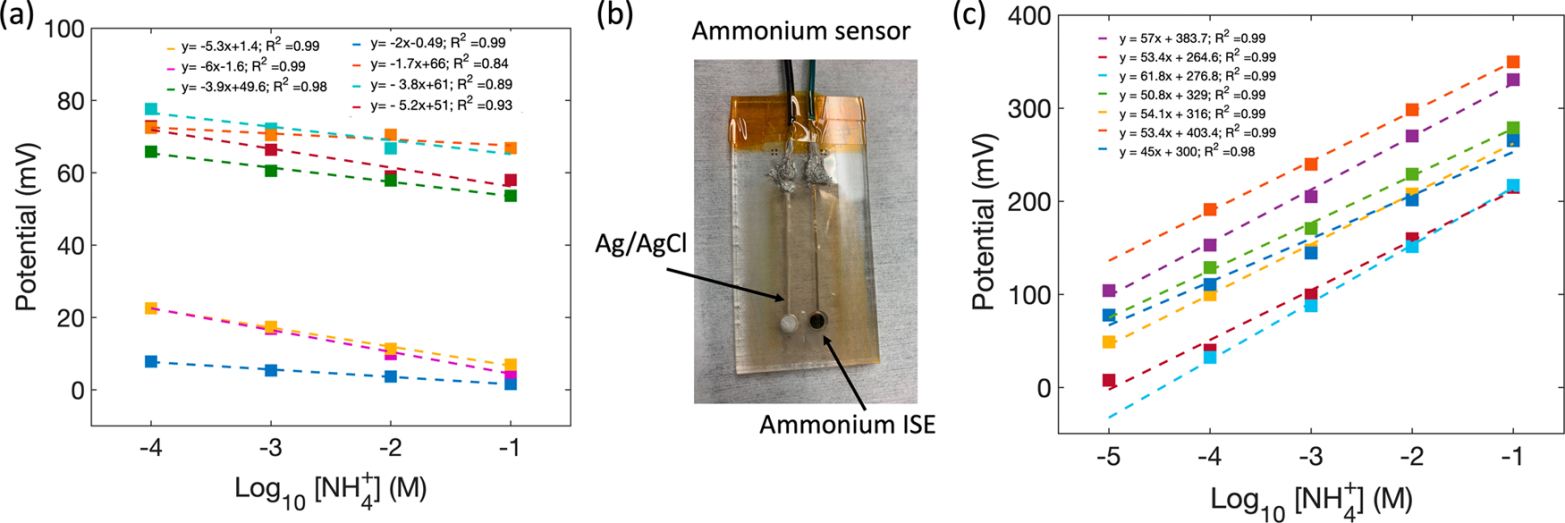
(a) Potential as a function of ammonium concentration for printed Ag/AgCl REs measured against a commercial Ag/AgCl RE. (b) Fully-printed ammonium sensor. (c) Sensitivity data for seven printed ammonium sensors from various batches. The average sensitivity is measured to be 53.6 ± 5.1 mV/decade.

The performance of printed REs was characterized by measuring the potential between a commercial Ag/AgCl double-junction RE and a printed RE. Figure 3a displays the potential response of seven printed REs measured in aqueous solutions with ammonium concentration varying from 0.1 to 100 mM. All printed REs showed low sensitivity with an average of −3.98 ± 1.66 mV/dec. The stability of the printed RE potential shown over 24 h in 100 mM NH_4_Cl solution is shown in Figure S3 of the Supporting Information.

### Fully Printed Sensors

The sensitivity of the fully printed ammonium sensors was characterized by measuring the potential between a printed ammonium ISE and a printed RE over time with changing ammonium concentrations. Figure 3b shows a fully-printed ammonium sensor. The sensitivities of printed ammonium sensors are shown in Figure 3c. Sensitivities for seven printed ammonium sensors in aqueous solution ranged from approximately 45 to 62 mV/dec. The average sensitivity was measured to be 53.6 ± 5.1 mV/decade and the *R*^2^ was 0.99. The linear range for these sensors is between 0.1 and 100 mM which is equivalent to 1.8 to 1804 ppm ammonium or 1.4 to 1400 ppm of nitrogen (NH_4_^+^-N).

### Testing in Soil

The aim of soil tests is to understand how the environmental factors introduced by soil might affect sensor performance. To capture a range of soil conditions, we tested the sensors in 3 different soils. This included plain sand (silicon dioxide, SiO_2_) to test sensor performance in an inert soil with little to no microbial activity, no organic matter, and very low ion concentration. The second and third soils were air-dried agricultural clay-rich and peatland soils, respectively, from the Sacramento San Joaquin Delta, California. These soils were chosen to test sensor functionality across different reactive soil mineral and organic matter content.

**Figure 4:**
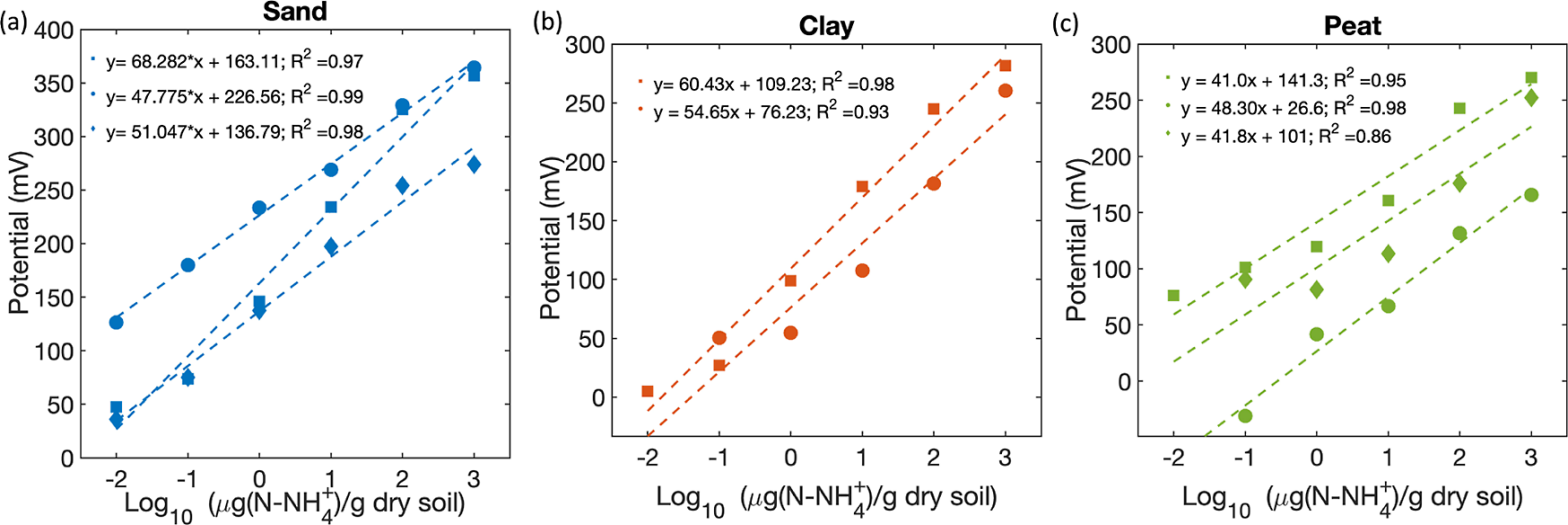
Linear response of fully printed ammonium sensors to variable ammonium ion concentration in saturated sand (a), clay (b), and peat (c) soils.

Ammonium sensors in sand showed a strong linear response with an average sensitivity of 55.7 ± 11 mV/dec and R^2^ of 0.98. The linear relationship between the output potential and the ammonium concentration in saturated sand is shown in Figure 4a. The average sensitivity of ammonium sensors in clay was 57.5 ± 4.1 mV/dec and *R*^2^ was 0.96 (Figure 4b). As shown in Figure 4c, ammonium sensors in peat exhibited an average sensitivity of 43.7 ± 4 mV/dec and *R*^2^ of 0.93, demonstrating high sensitivity and strong correlation to total soil ammonium for all three soil types. The sensors showed a comparable response in sand and clay soils. However, relatively low sensitivity and *R*^2^ were measured in peat soil. This might be due to damage in the sensing membrane. The sensors were first tested in sand and clay soils followed by measurements in peat. This means that sensors were subjected to several cycles of insertion, removal, and rinsing from the soil media. So, it is possible that the sensors were somewhat damaged by the time measurements in peat soil were started.

**Figure 5:**
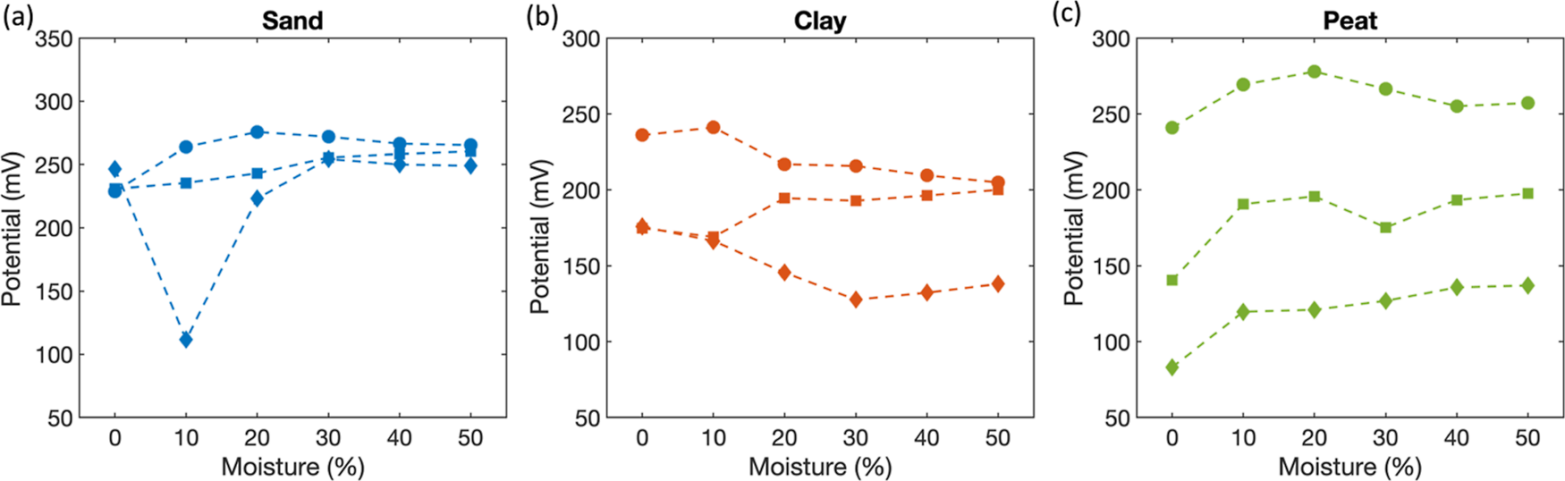
(a) Response of ammonium sensors under variable soil moisture content, ranging from 0 to 50% soil moisture by addition of 100 mM ammonium chloride solution in sand (a), clay (b), and peat (c) soils.

We also studied the effect of soil moisture on the performance of ammonium sensors in different soil types. Figure 5 shows the output potential of ammonium sensors as a function of the soil moisture content in sand, clay, and peat soils, while the ammonium concentration was kept constant at 100 mM. By adding a high concentration ammonium solution, any pre-existing ammonium in the soil would be negligible in comparison. Ideally, the sensors’ response should be a flat potential signal as the ammonium concentration was not varied. The sensors’ output response in sand with moisture content below 20% was unstable, with variations as large as 135 mV (Figure 5a). Beyond 20% soil moisture, the signal was stable resulting in constant potential as moisture content is varied. As shown in Figure 5b,c, the threshold moisture for clay and peat soil was in the 10–30% and 10–20% range, respectively. Potentiometric measurements require an ionic contact between the RE and ISE to allow the free movement of ions between the two electrodes. Hence, a threshold soil moisture is needed to support the flow of ions. It is evident from Figure 5 that the threshold value depends on the soil type. Overall, the results shown in Figures 4 and 5 indicate the promise of printed ammonium sensors for potential applications in soil media.

Soil variability can affect the performance of printed ammonium sensors by introducing inconsistencies in sensor readings due to differences in soil properties, moisture content, temperature, salinity, and sensor placement. Addressing these factors through calibration, strategic sensor deployment, and environmental management can help to improve sensor accuracy and reliability. We plan to implement these measures and study their effect on the ammonium sensor performance in soil media.

## Conclusions

We have demonstrated low-cost, fully printed ammonium sensors that can quantify the ammonium content in diverse soil environments. The printed ammonium sensors were obtained by pairing a printed RE and a printed ammonium ISE. The interaction of the ammonium ISM with ammonium ions resulted in a potential difference between the RE and ISE. Using this generated potential difference, the ammonium concentration in the soil could be determined. When paired with a glass RE, ammonium ISEs showed a sensitivity of 49.6 ± 3.6 mV/dec in solution. A printed RE with low sensitivity to ammonium was also developed using a reference membrane of PVB and NaCl. Fully printed ammonium sensors demonstrated a near-Nernstian sensitivity of 53.6 ± 5.1 mV/dec in an aqueous salt solution. Further, the sensors demonstrated a sensitivity of 55.7 ± 11, 57.5 ± 4.1, and 43.7 ± 4 mV/dec in the sand, clay, and peat soils, respectively. In addition, we demonstrated that the sensors were stable throughout the repeated tests in a variety of diverse environments, including an aqueous solution and sand, clay, and peat soils. The sensors shown here use fabrication techniques that are scalable and relatively low-cost compared to conventional electronics. Consequently, these could be distributed throughout the agriculture field at a high spatial density to monitor soil macronutrients.

## Supporting Information Available

Printed ammonium sensor’s response to nonchemical stimuli, current-reversal chronopotentiogram and open-circuit potential response, and drift response for a printed RE (PDF)
