# Supplementary material for: Printed Potentiometric Ammonium Sensors for Agriculture Applications
Source: ACS Omega. 2024 Nov 20;9(48):47453–60. doi: 10.1021/acsomega.4c05746 (PMC11618443; doi:10.1021/acsomega.4c05746)
Supplement: Supplementary file 1 — ao4c05746_si_001.pdf [file ao4c05746_si_001.pdf]

Supporting Information

Printed Potentiometric Ammonium Sensors for Agriculture Applications

Anju Toor,[*,†,‡] Payton Goodrich,[‡] Tyler Anthony,[¶] Claire Beckstoffer,[¶] Haeshini Jegan,[†] Whendee Silver,[¶] and Ana Claudia Arias[‡]

†*School of Materials Science and Engineering, Georgia Institute of Technology, Atlanta, Georgia, 30332, United States*

‡*Department of Electrical Engineering and Computer Science, University of California Berkeley, Berkeley, CA*

¶*Department of Environmental Science Policy and Management, University of California, Berkeley, Berkeley, CA*

E-mail: anju.toor@mse.gatech.edu

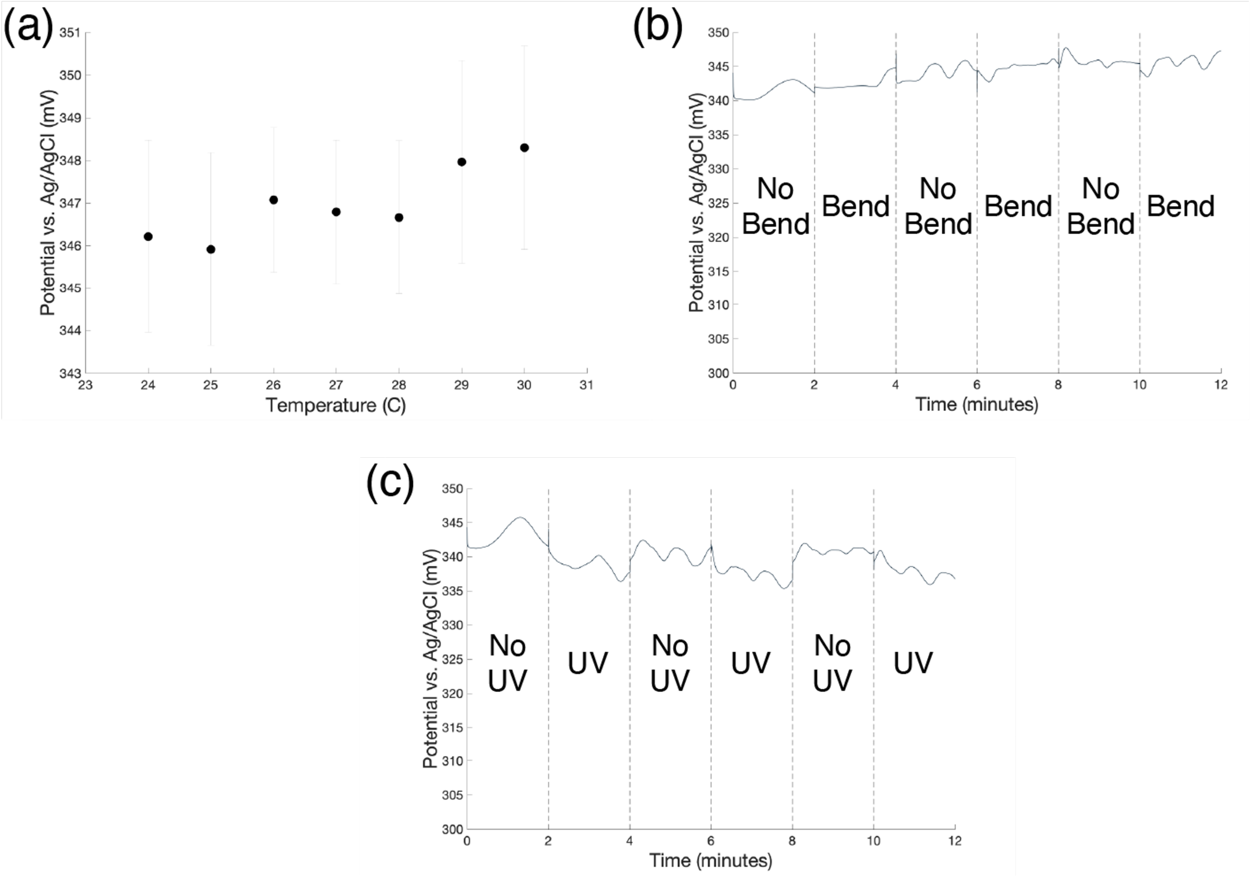

Figure S1: Printed ammonium sensor response to non-chemical stimuli. A printed ammonium sensor was submerged in 100 mM NH_4Cl solution and the open-circuit potential was measured. (a) Potential response to changes in solution temperature. (b) Potential response when the sensor is subject to a 30 degree bend. (c) Potential response to 405nm UV-light.

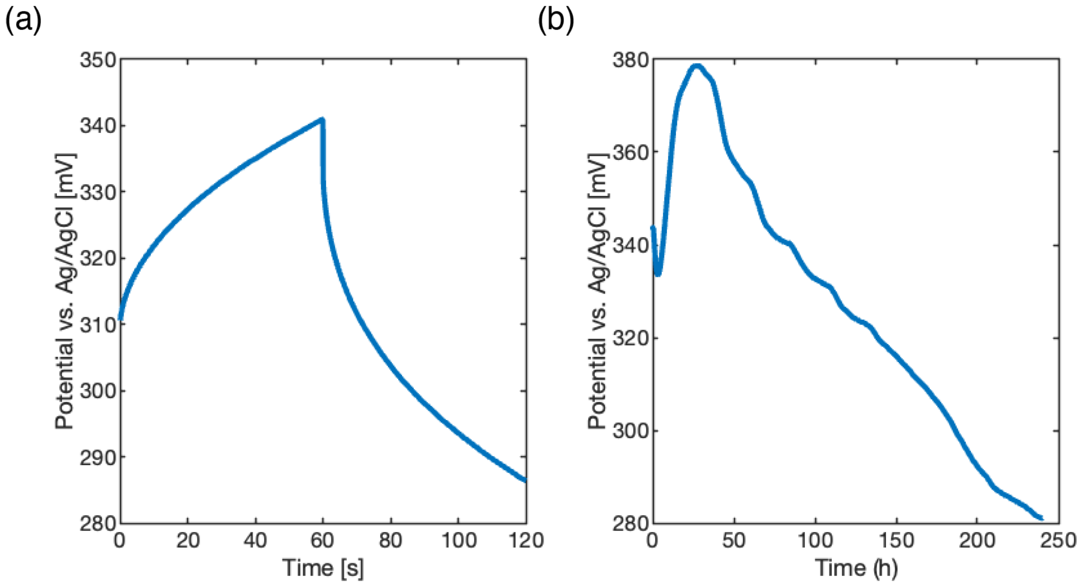

Figure S2: a) Current-reversal chronopotentiogram of an ammonium ISE recorded in 100 mM NH_4Cl. b) Open-circuit potential of the printed ammonium ISE vs. a commercial Ag/AgCl RE in 100 mM NH_4Cl.

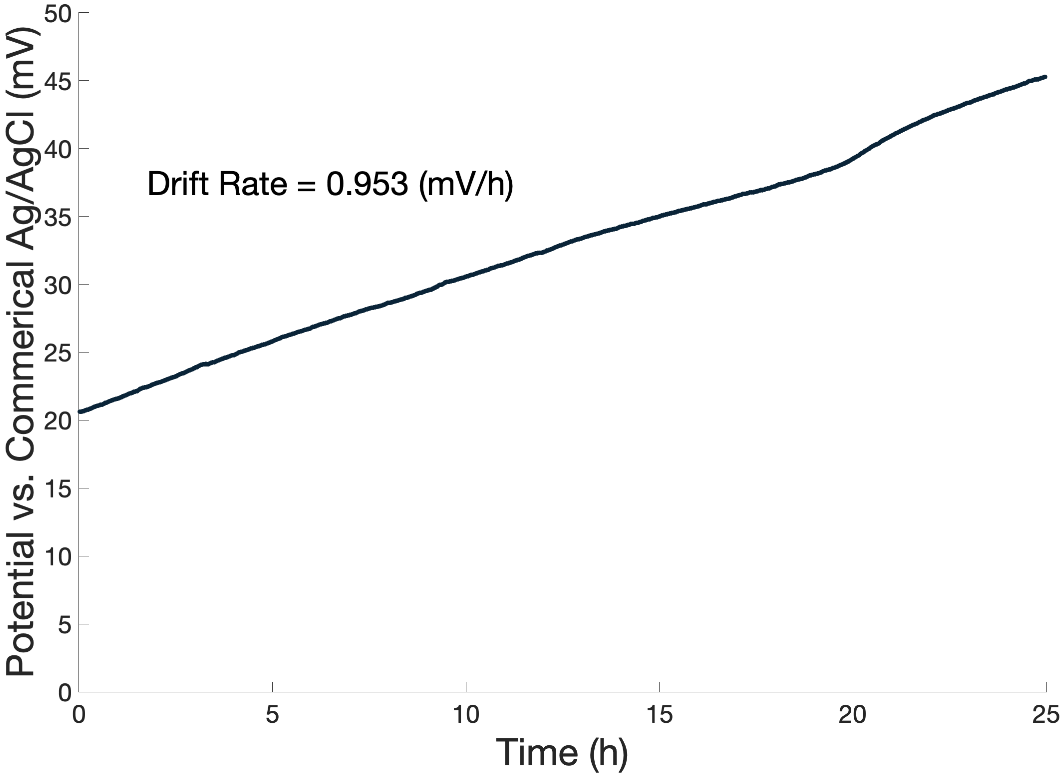

Figure S3: Printed reference electrode potential recorded in 100 mM NH_4Cl solution for 24 hours.
